# The effect of preoperative topical magnesium sulfate spraying in the oropharyngeal region on postoperative sore throat following gynecological laparoscopic surgery: a randomized clinical trial

**DOI:** 10.1186/s12871-025-02893-7

**Published:** 2025-01-08

**Authors:** Linxin Wang, Fangfang Li, Yuqing Liu, Xingyu Xiong, Qin Qiu, Guanglei Wang

**Affiliations:** Department of Anesthesiology, The Affiliated Hospital of Xuzhou Medical University, No. 99 Huaihai West Road, Quanshan District, Xuzhou, Jiangsu 221006 People’s Republic of China

**Keywords:** Postoperative sore throat, Magnesium, General anesthesia, Endotracheal intubation

## Abstract

**Background:**

Postoperative sore throat is a common complication following general anesthesia. This study aimed to investigate the impact of preoperative topical magnesium sulfate spraying in the oropharyngeal region on postoperative sore throat following gynecological laparoscopic surgery.

**Methods:**

The study included 58 patients scheduled for gynecologic laparoscopic surgery at Xuzhou Medical University Affiliated Hospital. Patients were randomly assigned to either the magnesium sulfate group or the control group, with 29 patients in each group. In the magnesium sulfate group, 25% magnesium sulfate was sprayed at a dose of 15 mg/kg on the mucous membrane of the pharynx and posterior wall of the larynx near the glottis using a laryngoscope under visual guidance during intubation; in the control group, an equal volume of normal saline was used instead. The primary outcome measure was the overall incidence of postoperative sore throat 48 h after surgery.

**Results:**

The overall incidence of postoperative sore throat in the magnesium sulfate group was lower than that in the the control group (20.0% vs. 66.7%, *P* < 0.001). At time points T1 (*P* < 0.001), T2 (*P* = 0.02), and T3 (*P* = 0.015), the incidence of postoperative sore throat in the the magnesium sulfate group was also lower than that in the the control group.

**Conclusion:**

This study confirmed that preoperatively spraying magnesium sulfate locally in the Oropharyngeal Region can reduce the frequency and severity of postoperative sore throat.

**Trial registration:**

The study was registered at Chictr.org.cn with the number ChiCTR2400087240 on 07/23/2024.

## Introdution

Postoperative sore throat (POST) is a prevalent complication following tracheal intubation under general anesthesia, which can result in postoperative discomfort and hinder eating, thereby delaying recovery [1]. Despite being self-limiting, POST remains among the top ten adverse outcomes associated with anesthesia [2]. The overall incidence of POST in adults ranges from 14.4–65% [3, 4]. In adults, POST typically peaks between 2 and 4 h after extubation. Although it usually subsides over time, its effects persist for up to 12 to 24 h, leading to reduced patient satisfaction post-surgery. Several risk factors contribute to the occurrence of POST including surgical factors such as site, position, and duration; patient-related factors like gender, age, airway condition, smoking history prior to surgery; and anesthesia-related factors encompassing tracheal tube size and material selection along with cuff shape and pressure management during intubation and techniques employed for extubation and sputum suction [5]. Anesthesiologists commonly employ strategies such as selecting appropriate endotracheal tubes with optimal cuff pressure levels while considering insertion methods and angles or implementing measures like preheating the catheter or utilizing traditional Chinese medicine acupuncture techniques alongside visual endotracheal tubes or pharmacological interventions aiming at reducing the incidence of POST [6]. Anesthesiologists used prevention and treatment methods for postoperative sore throat according to the patient’s own conditions in order to follow the latest guidelines for preoperative evaluation of patients undergoing noncardiac surgeryand reduce the length of hospital stay [7]. 

The head-down position is frequently utilized in gynecological surgery due to its facilitation of surgical field exposure and ease of operation. Prolonged head-down tilt can result in swelling of the face, conjunctiva, larynx, and tongue, thereby increasing the likelihood of postoperative upper airway obstruction. Patients undergoing lengthy surgeries in the head-down position may experience edema of the trachea and throat mucosa, consequently heightening the risk of POST. Gender represents one of the risk factors associated with POST. Females generally possess thinner tracheas and more delicate tracheal wall mucosa, rendering them more susceptible to developing POST [8]. 

Magnesium sulfate, an antagonist of the N-methyl-D-aspartate (NMDA) receptor, is currently one of the most efficacious drugs for the prevention and treatment of POST [9, 10]. Its mechanism of action involves peripheral NMDA receptor activation, which may induce pain in masticatory muscles, skin, and deep tissues. The anti-nociceptive effect of magnesium is achieved the through inhibition of calcium entry into cells by blocking NMDA receptors. Due to its ready ionization in solution, locally absorbed magnesium can be effectively utilized by surrounding tissues. Notably, NMDA receptors are present both centrally and peripherally; thus local administration of magnesium antagonizes noxious stimuli resulting from mucosal inflammation caused by tracheal intubation. Moreover, magnesium sulfate exhibits convergent anti-inflammatory properties and reduces traumatic edema and inflammation [11, 12]. Gargling, pump injection, and atomization inhalation are the main methods employed for administering magnesium sulfate to prevent POST. However, there has been no investigation on the use of local spraying with magnesium sulfate in preventing POST after gynecological laparoscopic surgery. Therefore, this study aims to explore the effects of preoperative local spraying with magnesium sulfate on sore throat following gynecological laparoscopic surgery.

## Methods

### Participants

This study was designed as a prospective, randomized, double-blind, single-center controlled clinical trial. It received approval from the Medical Ethics Committee of the Affiliated Hospital of Xuzhou Medical University and was registered in the Chinese Clinical Trial Registry(ChiCTR2400087240). Written informed consent was obtained from all participants prior to enrollment. The study has been reported in compliance with the Consolidated Standards of Reporting Trials (CONSORT) Guidelines, while the protocols were carried out adhering to the principles outlined in the Declaration of Helsinki.

The trial was conducted from July 2024 through September 2024. Inclusion criteria: (1) patients undergoing elective gynecological surgery; (2) American Society of Anaesthesiologists (ASA) grades I-II; (3) aged 18–64 years; (4) BMI of 18–30 kg/m². Exclusion criteria: (1) presence of preoperative oral ulcer, sore throat, throat mucosal injury, or pharyngitis; (2) occurrence of preoperative nausea and vomiting, cough, or dysphagia; (3) history of previous throat surgery; (4) Mallampati grade > 2; (5) patients with hypermagnesemia or hypersensitivity to magnesium prior to surgery; (6) recent respiratory infection before surgery; (7) hypoalbuminemia before surgery; (8) current smoking; (9) placement of a preoperative nasogastric tube; (10) severe cardiovascular, respiratory, liver, and kidney disfunction; (11) treatment with chronic use of calcium channel blockers or magnesium; (12) magnesium sulfate contraindications including acute intestinal bleeding, acute abdomen conditions, pregnancy, and lactation; (13) postoperative intubation was performed again; (14) multiple intubations performed twice or more times during the procedure; (15) durations less than 1 h or greater than 5 h for endotracheal tube placement were excluded from analysis; (16) patients requiring admission to the intensive care unit after surgery.

### Randomization and masking

The patients were randomly allocated into two groups: the control group (group C) and the magnesium group (group M) with an allocation ratio of 1:1 according to a computer-generated random number table. A nurse who was unaware of the trial details sealed the group assignments in sequentially numbered opaque envelopes, and the anesthesia nurse opened the envelopes and prepared the trial medications in syringes according to group assignment, with no differences in the appearance of the syringes or medications. Both magnesium sulfate spraying and normal saline spraying, as well as tracheal intubation, were performed by the same experienced senior anesthesiologist. Intraoperative indicators were recorded, followed by postoperative follow-up conducted by another blinded anesthesiologist with respect to grouping assignment, while patients remained unaware of their respective groups. Data analysis of trial results was performed by a statistician who remained unaware of group assignments until completion.

### Intervention

Upon entering the room, routine monitoring was conducted for ECG, BP, SpO_2_, and P_ET_CO_2_. Venous access to the upper limbs was established. The Ai anesthesia depth monitor was utilized to monitor anesthesia depth, while the TOF muscle relaxation monitor was used to assess muscle relaxation levels. Anesthesia induction involved a 5-minute preoxygenation period followed by administration of midazolam (0.05 mg/kg), etomidate (0.3 mg/kg), sufentanil (0.5 µg/kg), rocuronium (0.6 mg/kg), and subsequent tracheal intubation.

In the group M, 25% magnesium sulfate at a dose of 15 mg/kg was sprayed onto the mucosa of the pharyngeal region near the vocal cords and the posterior pharyngeal wall using a single-use ear, nose, and throat anesthesia spray device (hereafter referred to as the throat spray) (TUORen Medical Equipment Co., Henan, China) under visualization with a video laryngoscope (administration should be completed within 10 s before intubation). In the group C, an equal volume of normal saline was sprayed onto the same area under the same conditions using the the throat spray. The detailed procedures for drug administration are as follows: The laryngeal anesthesia tube was bent into a shape roughly similar to that of the tracheal tube before insertion, and the epiglottis was not provoked during spraying; epiglottis and the tongue root spray: 1/3, both sides of aryepiglottic fold spray: 1/3, the posterior pharyngeal wall spray: 1/3. The cuff pressure in endotracheal tube maintenance ranged from 22 to 26 cm H2O. Anesthesia maintenance included propofol infusion ranging from 4 to 12 mg/kg/h, remifentanil infusion ranging from 0.2 to 0.3 µg/kg/min, and sevoflurane inhalation maintaining MAC level at approximately 1.3. Throughout the procedure, depth of anesthesia index (AI index) values were maintained between 40 and 60 while blood pressure and heart rate were kept within ± 20% range compared to baseline values. Additional doses of rocuronium were administered based on results obtained from muscle relaxation monitoring to maintain TOF ratio at or above 0. Mechanical ventilation was conducted with a tidal volume of 6-8 ml/kg, an inspiratory/expiratory ratio of 1:1.5, a fraction of inspired oxygen (FiO_2_) of 60%, an oxygen flow rate of 2 L/min, and the respiratory rate was adjusted to maintain end-tidal carbon dioxide (PETCO2) levels between 35 and 45 mmHg. Administration of propofol and remifentanil ceased at the conclusion of the procedure. Extubation occurred once extubation criteria were met. Upon admission to the Post-Anesthesia Care Unit (PACU), patients were transferred back to their ward upon discharge from PACU.

### Outcomes

The primary outcome measure was the overall incidence of POST 48 h after surgery. The secondary outcome included the incidence of sore throat immediately after extubation (T1), at 2 h (T2), 6 h (T3), 12 h (T4), 24 h (T5), and 48 h (T6) after the operation. Sore throat pain scores were assessed immediately after extubation (T1), at 2 h (T2), 6 h (T3), 12 h (T4), 24 h(T5) and 48 h(T6) post-operation using a Numeric Rating Scale [NRS] [13] ranging from scale0(no pain) to scale10(worst pain). The occurrence rate of coughing during extubation. The incidence of nausea and vomiting within 48 h after surgery and the incidence of postoperative dysphagia were recorded. The study also evaluated the incidence of postoperative hoarseness immediately after extubation(T1), at T2(2 h post-op.), T3(6 h post-op.), T4(12 h post-op.), T5(24 h post-op.) and T6(48 h post-op.). Furthermore, the peak airway pressure was evaluated at 5 min, 30 min, 1 h, and 90 min following intubation. Additionally, The Quality of Recovery Scale-15 (QoR-15), was employed to assess the postoperative recovery quality at the 48-hour mark.

### Statistical analysis

The sample size was determined using PASS version 15.0, which was based on clinical practice observations and relevant literature research findings. In a previous study, the incidence of sore throat after gynecologic laparoscopy was 69% [14]. In addition, we performed a pilot experiment and showed a 60% reduction in the incidence of postoperative sore throat after magnesium sulfate spraying. Assuming an α level of 0.05, a power (1-β) of 90%, and accounting for a potential dropout rate of 10%, the calculated sample size was 29 per group, resulting in a total sample size of 58 participants.

Data analysis was performed using SPSS software version 26.0. The normality of data distribution was assessed using the Shapiro-Wilk test, while homogeneity of variance was examined using the Levene method. Normally distributed measurement data were presented as mean ± standard deviation, whereas non-normally distributed measurement data were expressed as median (M) and interquartile range (IQR), with comparisons conducted through the Mann-Whitney U test. Generalized estimating equation (GEE) analysis was employed to analyze repeated measurements at each time point within groups. Count data rates (%) were evaluated using either the chi-square test or Fisher’s exact test.

## Results

**Fig. 1:**
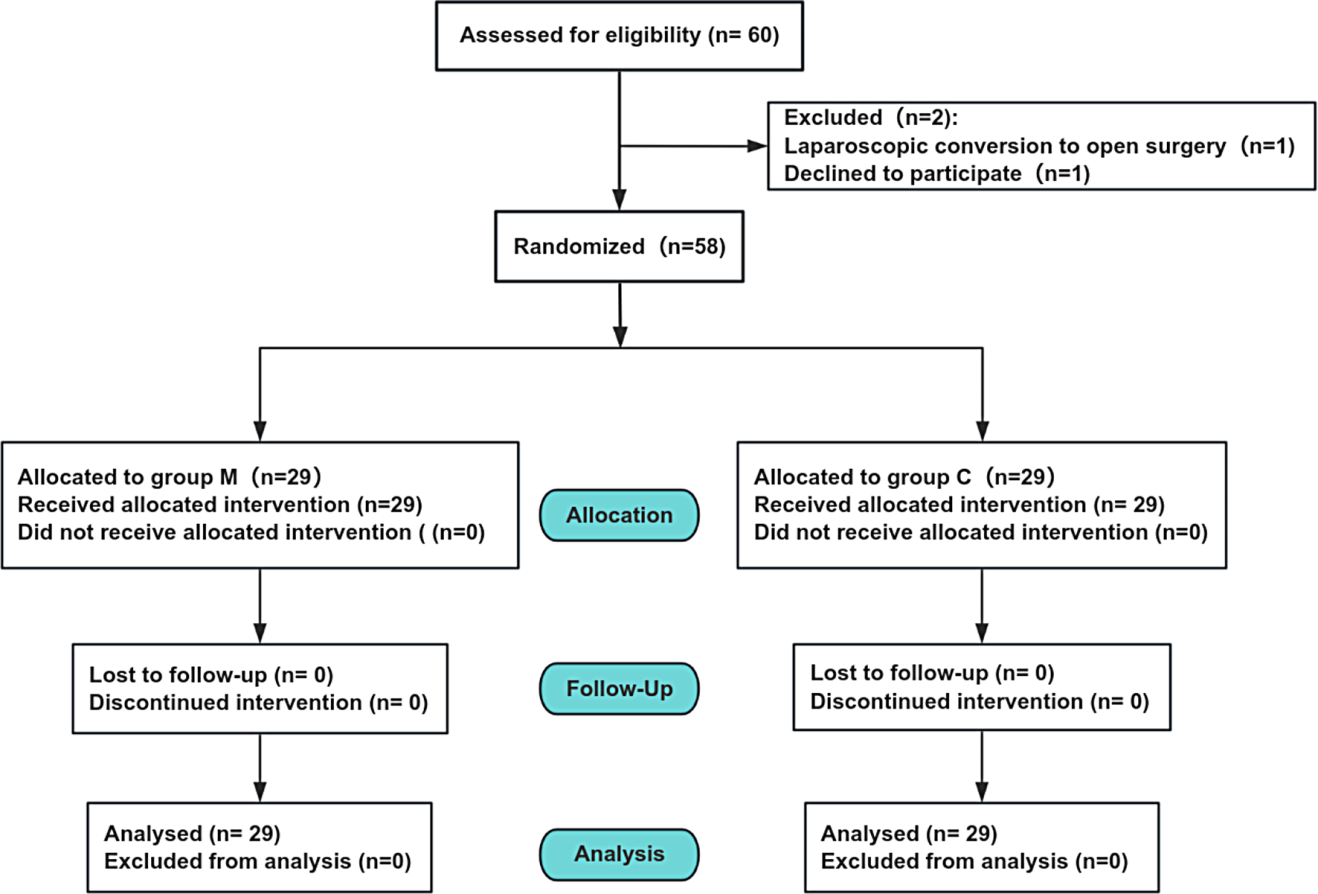
Study flow diagram

Between July 2024 and September 2024, we assessed for eligibility 60 patients. Two patients did not meet the criteria, All 58 participants successfully completed the study, ensuring that no exclusions were made for statistical analysis (Fig. 1).

The general conditions between the two groups were compared. There were no statistically significant differences in age, body mass index (BMI), preoperative albumin levels, preoperative serum magnesium ion concentration, postoperative serum magnesium ion concentration, preoperative and postoperative serum magnesium ion difference, intraoperative infusion volume, intraoperative urine volume, intraoperative remifentanil infusion volume, intraoperative atropine dosage, cuff pressure 5 min after intubation, 30 min after intubation, 1 h after intubation and 90 min after intubation (Table 1).

**Table 1:** Cohort characteristics

Variables	Control Group (*n* = 29)	Magnesium Group (*n* = 29)	*p*
Age (years)	41.47 ± 9.28	43.33 ± 9.65	0.448
Height (cm)	1.61 ± 0.05	1.61 ± 0.05	0.980
Weight (kg)	65.75 ± 8.7	62.03 ± 8.7	0.104
BMI (kg/m^2^)	25.26 ± 2.99	23.88 ± 3.26	0.093
Duration of tracheal intubation (min)	155.4 ± 47.9	137.9 ± 45.77	0.859
Preoperative serum albumin levels (g/dl)	47.023 ± 2.99	46.37 ± 3.37	0.433
Preoperative serum magnesium level (mmol/L)	0.81 ± 0.07	0.85 ± 0.07	0.116
Postoperative serum magnesium ion level (mmol/L)	0.8(0.77–0.82)	0.81(0.8–0.85)	0.286
The variation in serum magnesium ion levels before and after the surgical procedure (mmol/L)	−0.018 ± 0.03	−0.033 ± 0.047	0.128
Intraoperative fluid volume infusion (ml)	1000(1000–1250)	1000(1000–1250)	0.556
Intraoperative urine volume (ml)	100(75–105)	80(75–100)	0.078
Remifentanil (mg)	1.5(1.5–2)	1.63(1.19–2.25)	0.922
atropine (mg)	0(0–0)	0(0–0)	0.321
Cuff pressure (mmHg)			
5 min after intubation	24(24–24)	24(22–24)	0.088
30 min after intubation	24(24–24)	24(22–24)	0.318
1 h after intubation	24(24–24)	24(22–24)	0.505
90 min after intubation	24(24–24)	24(24–24)	0.985

BMI = body mass index. Values are shown as percentages, mean ± standard deviation, or median (interquartile range)

Comparison of postoperative sore throat between the two groups. The overall incidence of POST in the group M was lower than that in the group C (20.0% vs. 66.7%, *P* < 0.001). At time points T1 (*P* < 0.001), T2 (*P* = 0.02), and T3 (*P* = 0.015), the incidence of POST in the group M was also lower than that in the group C. At time points T4 (*P* = 0.095), T5 (*P* = 0.667), and T6 (*P* = 1.000), there was no statistically significant difference in the incidence of POST between the group M and the group C (Table 2).

The interaction between group and time was examined using generalized estimating equations. The independent effect analysis of time revealed a significant downward trend in the POST NRS score for group C, with statistically differences observed at T1 and T3 (*P* = 0.046). Additionally, there were statistically significant differences between T1 and T4 (*P* = 0.009), T1 and T5 (*P* = 0.001), as well as T1 and T6 (*P* = 0.004). In contrast, no significant differences were found in the POST NRS score at each time point for group M. Furthermore, separate effect analysis between groups demonstrated that the POST NRS scores differed significantly between the two groups at T1(*P* < 0.001), T2 (*P* = 0.014), and T3(*P* = 0.013) (Table 2).

**Table 2:** Incidence and severity of postoperative Sore Throat

Variables	Control Group (*n* = 29)	Magnesium Group (*n* = 29)	χ²/Z	*p*
Sore throat				
Overall incidence	20(66.7%)	6(20.0%)	13.303	< 0.001^a^
0 h postoperatively(T1)	18(60.0%)	4(13.3%)	14.067	< 0.001^a^
2 h postoperatively(T2)	12(40.0%)	4(13.3%)	5.455	0.02^a^
6 h postoperatively(T3)	11(36.7%)	3(10.0%)	5.963	0.015^a^
12 h postoperatively(T4)	8(26.7%)	3(10.0%)	2.783	0.095
24 h postoperatively(T5)	4(13.3%)	2(6.7%)	0.185	0.667
48 h postoperatively(T6)	3(10.0%)	2(6.7%)	0.000	1.000
NRS of sore throat^b^				
0 h postoperatively(T1)	1(0–2)	0(0–0)	−3.822	< 0.001^a^
2 h postoperatively(T2)	0(0–1)	0(0–0)	−2.448	0.014^a^
6 h postoperatively(T3)	0(0–1)^c^	0(0–1)	−2.497	0.013^a^
12 h postoperatively(T4)	0(0–0)^c^	0(0–0)	−1.684	0.092
24 h postoperatively(T5)	0(0–0)^c^	0(0–0)	−0.881	0.378
48 h postoperatively(T6)	0(0–0)^c^	0(0–0)	−0.524	0.600

^a^ Compared Magnesium Group with Control Group, *P* < 0.05

^b^ NRS of sore throat were compared in generalized estimating equations between and within groups (*P* for group < 0.001, *P* for time < 0.001, *P* for interaction = 0.019; Waldχ² for groups = 11.514, Waldχ² for time = 20.016, Waldχ² for interaction = 13.534)

^c^*P* < 0.05 for each time point vs. T1 within Magnesium group

Other conditions were compared between the two groups. There was no statistically significant difference in the incidence of coughing after extubation between the two groups (*P* = 0.084). The incidence of postoperative nausea and vomiting did not differ significantly between the two groups (*P* = 0.1). Similarly, there was no significant difference in the incidence of postoperative dysphagia between the two groups (*P* = 1.000). However, at T3, group M exhibited a lower incidence of hoarseness compared to group C (*P* = 0.037). No significant differences in hoarseness were observed between the two groups at T1 (*P* = 0.222), T2 (*P* = 0.091), T4 (*P* = 0.417), T5 (*P* = 1.000), and T6 (*P* = 0.704). Furthermore, peak airway pressure at 5 min (*P* = 0.024) and 30 min (*P* = 0.041) after intubation was significantly lower in group M than in group C; however, There was no significant difference in peak airway pressure between the two groups at 1 h after intubation (*P* = 0.166) and 90 min after intubation (*P* = 0.817). Additionally, there was no statistically significant difference in the QoR-15 score at 48 h after operation between the two groups (*P* = 0.239)(Table 3).

The incidence of adverse reactions was compared between the two groups. The incidence of adverse reactions was 0 in both groups (all *P* = 1), such as attenuated or abolished tendon reflexes, reduced respiratory rate, facial flushing, perspiration, xerostomia, diarrhea, or hypersensitivity reaction (Table 4).

**Table 3:** Other secondary outcome measures

Variables	Control Group (*n* = 29)	Magnesium Group (*n* = 29)	*p*
Coughig during extubation	0(0–0)	0(0–0.25)	0.084
Postoperative nausea and vomiting(%)	23(76.7%)	17(56.7%)	0.100
Postoperative dysphagia(%)	1(3.3%)	2(6.7%)	1.000
Postoperative hoarseness(%)			
0 h postoperatively(T1)	25(83.3%)	21(70.0%)	0.222
2 h postoperatively(T2)	24(80.0%)	18(60.0%)	0.091
6 h postoperatively(T3)	21(70.0%)	13(43.3%)	0.037^a^
12 h postoperatively(T4)	12(40.0%)	9(30.0%)	0.417
24 h postoperatively(T5)	7(23.3%)	7(23.3%)	1.000
48 h postoperatively(T6)	5(16.7%)	3(10.0%)	0.704
Peak airway pressure(cmH_2_O)			
5 min after intubation	15(13.75–18)	14(13–15)	0.024^a^
30 min after intubation	19.5(18–22.25)	17(15–21.75)	0.041^a^
1 h after intubation	18.8 ± 3.010	20.3 ± 5.25	0.166
90 min after intubation	15(13.75–18)	14(13–20.25)	0.817
The QoR-15 score at 48 h post-surgery	112.97 ± 16.60	118.47 ± 19.09	0.239

Values are shown as percentages, mean ± standard deviation, or median (interquartile range)

^a^ Compared Magnesium Group with Control Group, *P* < 0.05

**Table 4:** The incidence of adverse reactions in the two groups

adverse reactions	Control Group (*n* = 29)	Magnesium Group (*n* = 29)	*P*
Tendon reflexes were reduced or absent	0	0	1.00
Decreased respiratory rate	0	0	1.00
Facial flushing	0	0	1.00
Sweat out	0	0	1.00
xerostomia	0	0	1.00
diarrhea	0	0	1.00
Allergic reaction	0	0	1.00

## Discussion

Various preoperative strategies are available for the prevention and management of POST, with pharmacological interventions being the primary approach.These pharmacological treatments include nonsteroidal anti-inflammatory drugs (NSAIDs), corticosteroids, local anesthetics, N-methyl-D-aspartate (NMDA) receptor antagonists, among others. Numerous studies have demonstrated the efficacy of these drugs in managing POST, although the effectiveness of lidocaine remains controversial. The irritation caused by lidocaine solvents may potentially increase the incidence of POST [15]. Magnesium sulfate, an NMDA receptor antagonist, has been associated with the lowest incidence of POST according to a systematic review. The review indicates that topical magnesium treatment has the highest potential to alleviate POST within 24 h post-surgery (low evidence quality) [9]. 

Topical spraying of medications in the pharyngeal area is a commonly employed preoperative method for preventing POST. This technique often involves using a localized spraying device to administer the medication in an aerosolized mist to the oropharynx near the vocal cords [16–19]. Research by Doyeon Kim et al. has demonstrated that preoperative prophylactic spraying of benzocaine hydrochloride onto the cuff of the endotracheal tube and the pharyngeal area can reduce the incidence and severity of POST within 12 h after surgery [16]. A randomized controlled trial has shown that spraying ropivacaine in the pharyngeal area can decrease both the incidence of POST and the cough rate during extubation [17]. A systematic review has indicated that topical spraying of flurbiprofen axetil in the pharyngeal region can lower the occurrence of POST [18]. Furthermore, research by J Lin et al. found that topical spraying of lidocaine in the pharyngeal area is effective in reducing POST incidence [19]. Although there is substantial research on drug spraying, there are no published studies on the topical spraying of magnesium sulfate. This study provides evidence that topical spraying of magnesium sulfate in the pharyngeal area is also effective for the prevention and treatment of POST.

**Fig. 2:**
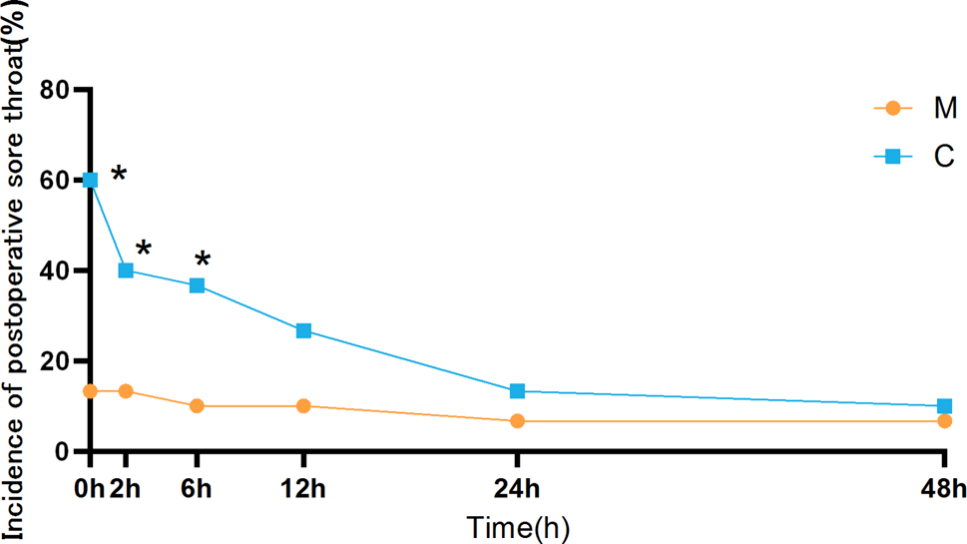
The incidence of postoperative sore throat in the two groups at each time period

*P < 0.05 compared to group M.

The overall occurrence of POST in the control group was approximately 66.7%, which is consistent with previous research findings [3, 4]. Preoperative local spraying of magnesium sulfate in the oropharynx can effectively reduce the occurrence of POST and its effect lasts for approximately 6 h, without any associated adverse reactions related to magnesium sulfate. Furthermore, preoperative local application of magnesium sulfate in the oropharynx can also alleviate the severity of POST, with its effects lasting for about 6 h. The duration of the effect is approximately 6 h. A possible reason is that POST is self-limiting, the incidence of POST in the group C showed a decreasing trend, with significant improvement observed at 6 h postoperatively (Fig. 2). Several studies have demonstrated that different applications of magnesium can effectively decrease both the occurrence and severity of POST. Jin Ha Park et al.‘s non-inferiority trial demonstrated that the efficacy of continuous administration of magnesium sulfate via infusion in reducing the incidence of POST was comparable to that of dexamethasone [20]. Teymourian et al. revealed that gargling with magnesium sulfate can decrease the occurrence of POST [21]. Wang Mizhou [22] and Yadav [23] et al. illustrated that aerosol inhalation of magnesium sulfate could lower the incidence of POST and airway pressure. Hale Borazan et al. showed that preoperative administration of magnesium lozenges reduced both the frequency and severity of POST [24]. 

The study also revealed that preoperative local administration of magnesium sulfate in the throat effectively reduces the incidence of postoperative hoarseness after 6 h. Furthermore, it has been observed to decrease peak airway pressure at both 5 min and 30 min following intubation. A systematic review has demonstrated that magnesium usage is associated with the lowest occurrence of hoarseness and holds a likelihood of 73.63% for being the optimal drug in preventing postoperative hoarseness [9]. The administration of magnesium sulfate demonstrates convergent, anti-inflammatory, and detumescent properties in the management of traumatic edema and inflammation. In clinical practice, magnesium sulfate is commonly utilized for local edema to facilitate detumescence. In order to enhance visibility and facilitate surgical manipulation during gynecological laparoscopy, the Trendelenburg position is commonly used. Preoperative topical spraying of magnesium sulfate to the pharyngeal area can alleviate intraoperative pharyngeal edema, thereby reducing airway pressure during the procedure.

The strengths of this study are the following. Firstly, local spraying is a new application method of magnesium sulfate. Secondly, compared with intravenous infusion, this method has the advantages of less dosage, better targeting, and reducing the risk of potential magnesium ion toxicity. In addition, magnesium sulfate has a bitter taste, so compared with garbling and aerosol inhalation of magnesium sulfate, local spray of magnesium sulfate after induction can improve the patient’s comfort and cooperation, reduce the preoperative preparation time, reduce the difficulty of application, and can cross the tongue base and be more accurate.

This study extends the topical application of magnesium sulfate. Future research should explore the comparison of different effects of magnesium sulfate in multiple ways for postoperative sore throat. At the same time, it also provides a new idea for the application of other drugs for postoperative sore throat. This study has the limitation. it is a single-center randomized controlled trial with a relatively small sample size, which limits the generalizability and external validity of the findings; therefore, further large-scale studies are necessary.

## Conclusions

In summary, preoperative spraying of 25% magnesium sulfate in the throat reduced the overall incidence and severity of POST after surgery, as well as the incidence and severity of POST at extubation, 2 h postoperatively, and 6 h postoperatively. Additionally, this method also decreases the incidence of postoperative hoarseness and peak airway pressure after intubation.

## Data Availability

No datasets were generated or analysed during the current study.

## Abbreviations

POST: Postoperative sore throat
NMDA: N-methyl-D-aspartate
ASA: American Society of Anaesthesiologists
BMI: Body mass index
ECG: Electrocardiography
SpO_2_: Blood oxygen saturation
BP: Blood pressure
P_ET_CO_2_: End-Tidal Carbon Dioxide Pressure
PACU: Post-Anesthesia Care Unit
AI index: Depth of anesthesia index
